# Restricted morphological changes in infrapatellar fat pad during walking is revealed as a dynamics feature in symptomatic knee osteoarthritis

**DOI:** 10.1007/s10396-025-01569-6

**Published:** 2025-09-12

**Authors:** Yosuke Ishii, Miharu Sugimoto, Akinori Nekomoto, Atsuo Nakamae, Kexin Zhu, Takato Hashizume, Kohei Matsumura, Yuko Nakashima, Makoto Takahashi, Nobuo Adachi

**Affiliations:** 1 Department of Bio-Environmental Adaptation Sciences, Graduate School of Biomedical and Health Sciences, Hiroshima University, 1-2-3 Kasumi, Minami-Ku, Hiroshima, 734-8551 Japan; 2 Department of Biomechanics, Graduate School of Biomedical and Health Sciences, Hiroshima University, Hiroshima, Japan; 3 Department of Orthopaedic Surgery, Graduate School of Biomedical and Health Sciences, Hiroshima University, Hiroshima, Japan; 4 Department of Sports Medical Center, Hiroshima University Hospital, Hiroshima, Hiroshima Japan

**Keywords:** Dynamic ultrasound, Gait, Infrapatellar fat pad, Morphological change, Symptomatic knee osteoarthritis

## Abstract

**Purpose:**

Knee osteoarthritis (OA) is symptomatic, especially in terms of motion during activities of daily living. The infrapatellar fat pad (IFP) has a buffering function, owing to morphological changes within the knee joint, whereas poor morphological change in the anterior space of the IFP is often observed in symptomatic knee OA. This study aimed to investigate the correlation between morphological changes in the anterior space of the IFP during walking and symptoms in patients with knee OA.

**Methods:**

Twenty-six patients with knee OA (OA group) and 11 healthy volunteers (control group) participated in this study. Ultrasonography revealed the IFP thickness in the anterior space during static and dynamic evaluations in the supine and walking positions. The waveform of the IFP during walking was constructed with a continuance value of the IFP in video mode. Moreover, it identified the difference in IFP between maximum and minimum values on the waveform as morphological change in IFP (ΔIFP). A three-dimensional motion analysis system was used to calculate the kinetics and kinematics of walking. The OA group underwent clinical evaluation using the Knee Injury and Osteoarthritis Outcome Score (KOOS).

**Results:**

ΔIFP in the OA group was smaller than that in the control group, whereas there was no significant difference in supine IFP thickness. Moreover, there was a significant positive correlation between ΔIFP and KOOS-pain in the knee OA group, but not in terms of other parameters of IFP, kinetics, and kinematics.

**Conclusions:**

Patients with symptomatic knee OA exhibit restricted morphological change in IFP during walking.

## Introduction

Patients with knee osteoarthritis (OA) suffer from various symptoms underlying a complex disease [1]. The primary symptom is pain during loading stress, which occurs while standing and walking during daily activities. This indicates that mechanical loading is involved and partly describes the complex disease [2], but the detailed mechanism underlying the mechanopathology remains unknown.

The infrapatellar fat pad (IFP) includes blood vessels and nerve fibers [3–5], which are involved in knee pain. The IFP has been dominantly evaluated under static conditions using magnetic resonance imaging (MRI) [6, 7]. It should be noted that the IFP acts as a buffer during mechanical load and adapts to a variety of knee motions within the knee joint, owing to flexible morphological changes [8–10]. Thus, static evaluation of IFP may provide only limited information about the mechanopathology. In particular, restricted morphological changes in the IFP during motion are often observed in symptomatic patients with knee OA, whereas involvement of IFP morphological information remains controversial [11–14].

Ultrasonography can be used to evaluate the IFP under several conditions, with dynamic evaluation being able to capture morphological changes in the knee structure during walking [15, 16]. The IFP in knee OA often shows pathological changes in fibrogenesis and dysfunction of shock absorption [17, 18], and restricted morphological changes are often observed in symptomatic patients [19]. Based on these previous studies, the morphological changes in the IFP observed during dynamic ultrasound evaluation could be a promising parameter for assessing buffer dysfunction during mechanical load and adaption, and offer key information about elucidating the mechanism of symptomatic knee OA.

Some studies have focused on the anterior interval space, which is located in the tibial tuberosity around the proximate tibia [7, 20, 21]. They investigated the association between knee kinematics and changes in IFP shape using three-dimensional models. During knee extension, the IFP was shown to move in an anterior-inferior manner toward the tibia in the anterior space [7, 22]. Moreover, the movement of the IFP was smaller in knee OA patients when compared with healthy volunteers [22]. Therefore, morphological changes in the anterior space of the IFP may be involved in knee OA pathology and help us understand the mechanisms of knee pain in patients with knee OA.

This study aimed to investigate the correlation between morphological changes in the anterior space of the IFP during walking and symptoms in patients with knee OA. We hypothesized that knee OA is involved in restricted morphological changes in the anterior space of the IFP during walking, and that the correlation with symptoms would be better detected via dynamic evaluation than via static evaluation performed in the supine position.

## Materials and methods

### Participants

Between September 2023 and April 2024, we recruited 26 symptomatic patients visiting our out-patient clinic with radiologically diagnosed unilateral or bilateral medial knee OA (OA group: 11 females; mean age: 63.5 ± 9.8 years) and eleven healthy volunteers (control group: 5 females; mean age: 59.7 ± 8.4 years). All the participants were selected based on their ability to walk smoothly without a cane or support device. For patients with bilateral knee OA, the knee with more severe and intense pain was selected as the index limb, and healthy volunteers were randomly assigned. Patients with symptomatic knee OA had persistent knee pain most days over the last 3 months. Exclusion criteria included a history of orthopedic surgery in the index knee, neurological disease, trauma, or corticosteroid injection within the previous month. All the patients were asked to make an effort to use minimum medication. Demographic data are outlined in Table 1.

**Table 1:** Demographic data of the participants

	Knee OA	Control	*p*-value
Knees	26	11	
KL grade (I, II, III, IV)	2, 10, 9, 5		
Sex (M: F)	15 : 11	6 : 5	0.85
Age (years)	63.5 ± 9.8	59.7 ± 8.4	0.26
BMI (kg/m²)	24.3 ± 3.5	24.0 ± 4.0	0.79

M: F, male: female; BMI, body mass index; OA, osteoarthrosis; KL, Kellgren-Lawrence.

Values are shown as mean ± standard deviation. The significance difference shows *p* < 0.05.

This study was approved by our institution’s ethics department in accordance with the Declaration of Helsinki (E2021-2498-02). Informed consent was obtained from all the patients and healthy volunteers.

### Gait analyses

**Fig. 1:**
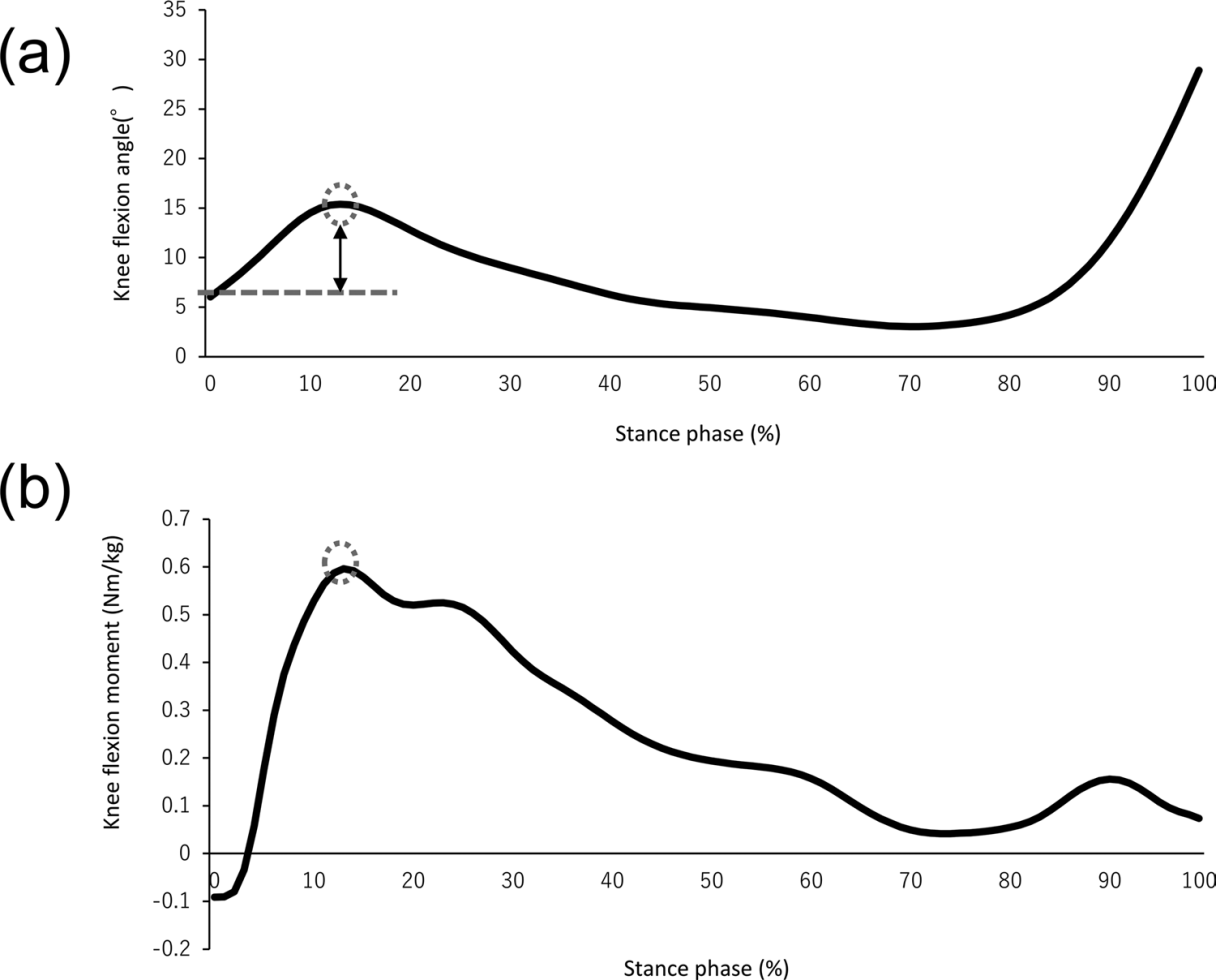
Waveforms of kinetic and kinematic parameters during walking Representative waveforms of the knee flexion angle (**a**) and knee flexion moment (**b**) during walking in a patient with knee OA. The timing of the peak values within the stance phase is indicated by the dotted mark. The dotted line and double arrow show the flexion angle at initial contact, and the range of flexion that is calculated as the difference in the flexion angle between the initial contact and the maximum point in the early stance phase

Kinematic and kinetic data of the participants were obtained using 16 cameras (Vicon Motion Systems) and eight force plates (AMTI, Watertown, Mass) with sampling rates of 100 and 1000 Hz, respectively. Reflective markers were attached to the anatomical lower landmarks based on the model of the plug-in-gait lower body (Vicon Motion Systems). Comfortable walking was performed, and this trial was repeated twice with each participant after several practice sessions to get accustomed to the environment. A single-stance phase of the gait cycle, including heel contact to toe-off, was precisely based on the ground reaction force exceeding a threshold of 10 N. Knee angle and moment were systematically calculated using the manufacturer’s tool (Nexus 1.8.5). Primary outcomes were knee kinetics and kinematics in the sagittal plane, knee flexion angle, and peak knee flexion moment. Range of flexion was calculated as the difference in the flexion angle between the initial contact and maximum point in the early stance phase on the single stance phase (Fig. 1). Eventually, the data were normalized to 101 data points for comparison with different trial durations (Fig. 1). Furthermore, the gait speed was estimated from heel markers, and these processing procedures were performed using MATLAB R 2020a (MathWorks). The average value was adopted as the representative data using a single gait cycle in each trial.

### Ultrasonography evaluation of IFP

**Fig. 2:**
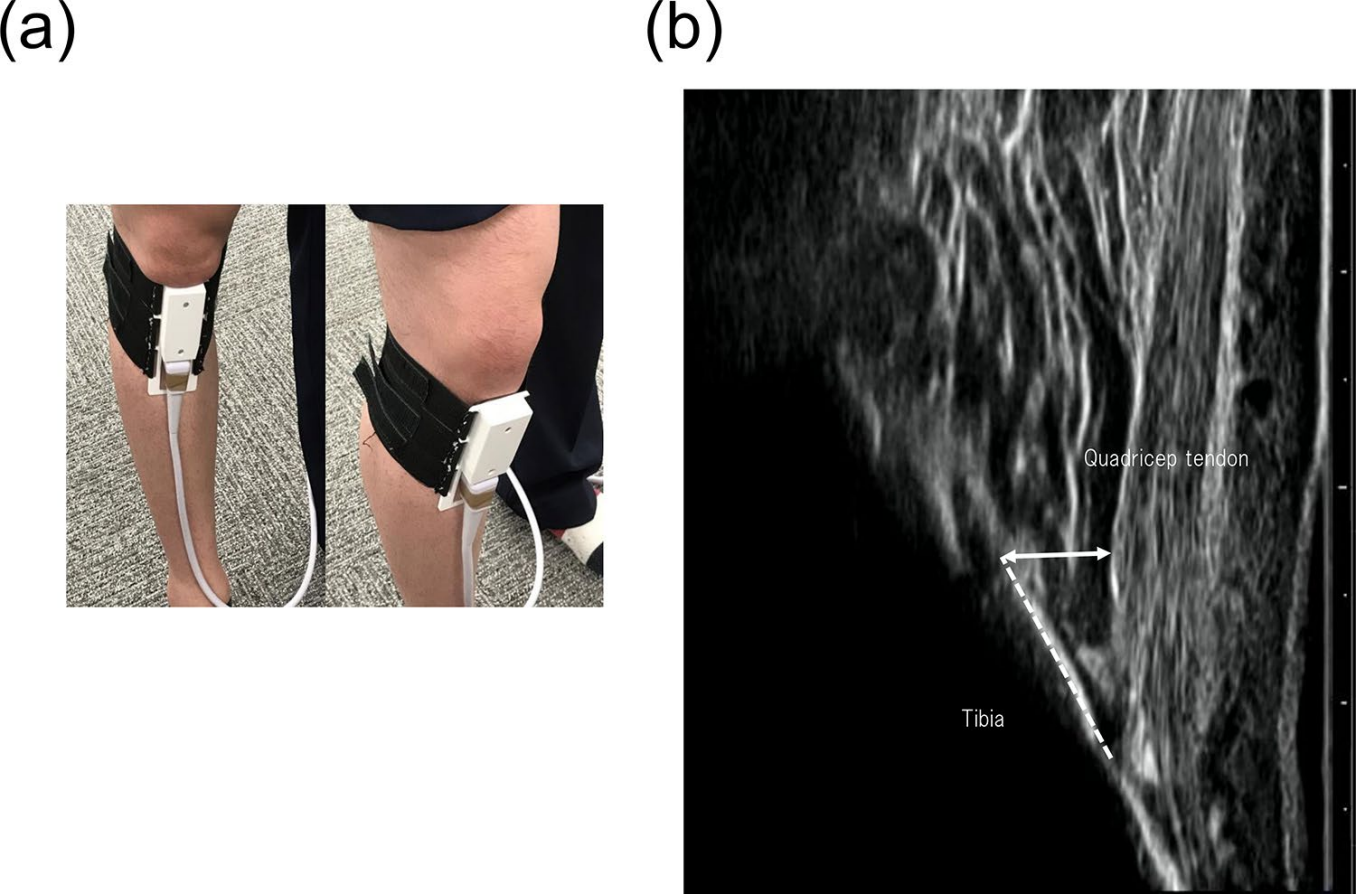
Imaging of the infrapatellar fat pad and definition Transducer placement (**a**) and representative ultrasound images (**b**). The dotted line shows the reference line on the tibia, which is 10 mm along the origin of tenderness in the quadriceps on the proximal tibia. The double arrow indicates the thickness of the IFP and perpendicular distance between the proximal tibial line and patellar tendon. IFP, infrapatellar fat pad

**Fig. 3:**
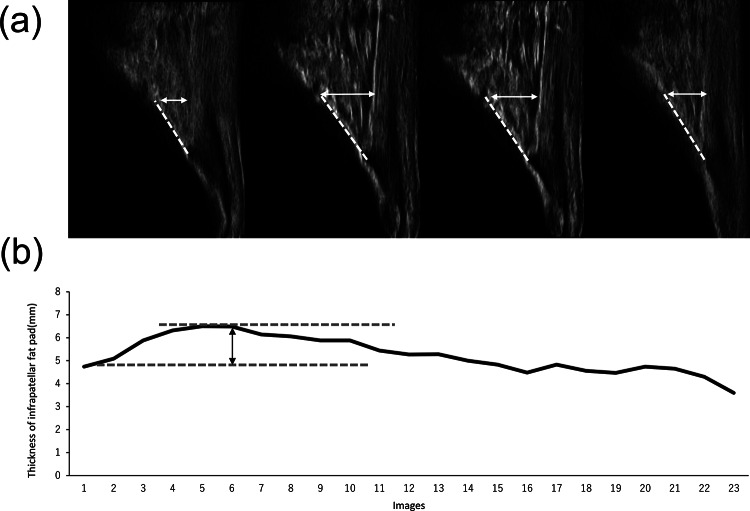
Reprehensive case with morphological change in IFP Representative images of the IFP in the stance phase while walking in approximate sequences of 20 frames (**a**). These images (gait cycle) are from frames 1 (1%), 6 (26%), 14 (66%), and 20 (96%), respectively. The continuous IFP values were consistent and showed a waveform (**b**). The double arrow indicates the morphological change in the IFP as ΔIFP, calculated as the difference between maximum and minimum values under the two dotted lines

**Fig. 4:**
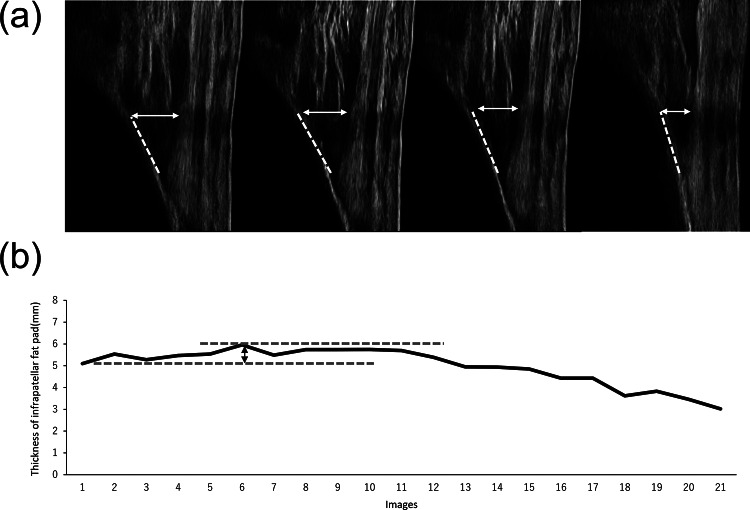
Reprehensive case with a restricted morphological change in IFP Representative images of the IFP in the stance phase while walking in approximate sequences of 20 frames (**a**). These images (gait cycle) are from frames 1 (1%), 6 (28%), 14 (69%), and 18 (90%), respectively. The continuous IFP values were consistent and showed a waveform (**b**). The double arrow indicates the morphological change in IFP as ΔIFP, calculated as the difference between maximum and minimum values under the two dotted lines

The IFP was evaluated using ultrasonography (SNiBLE; KONICA MINOLTA, Japan) with a linear-array prototype transducer (3–11 MHz). The participants were initially placed in the supine position, and the transducer was longitudinally placed on the patellar tendon with the inferior margin of the patella and their knees fully extended. The transducer was adjusted to depict the anterior interval of the IFP with a fibrillar pattern of the patellar tendon and optimal visualization of the distinct border between the patellar tendon and the IFP edge on the images, based on a previous study [21]. Eventually, the transducer, was secured using a special brace with a flexible band, and static measurements of the IFP were obtained (Fig. 2). Dynamic evaluation was performed to detect the behaviour of the IFP during walking, recorded in video mode at a sampling rate of 30 Hz, and synchronised with a motion analysis system. The IFP thickness was quantified as the perpendicular distance from a specified point on the anterior tibia positioned 10 mm proximal to the patellar tendon insertion to the inferior surface of the patellar tendon on the images. This calculation was performed using the Kinovea software (v0.8.15). A comprehensive dataset, including around 20 images, was acquired per trial for construction of an IFP waveform. Maximum and minimum values on the IFP waveform were detected, and morphological changes in the IFP (ΔIFP) were shown as the difference in IFP value from two points in each participant (Figs. 3 and 4). Moreover, the IFP waveform was also normalized to 101 data points. On ensuring reliability, a previous study confirmed moderate to high reliability using the same techniques [23].

### Assessment of IFP using MRI

MRI (Ingenia 3.0 T, Philips) was performed within 3 months of the first visit to our clinic. The evaluation was performed with the patient in the supine position with mild knee flexion. The IFP area in the patellofemoral joint was captured on a sagittal image, including the side that enabled visualization of the clearest patellar tendon.

### Clinical outcome assessment

Knee Injury and Osteoarthritis Outcome Scores (KOOS) were used to evaluate the course of knee injury and treatment outcomes. A high value indicated a favorable condition in the participant’s knee. The KOOS includes five subscales, consisting of symptoms (KOOS-S), pain (KOOS-P), activities of daily living (KOOS-A), sports/recreation (KOOS-Sp), and quality of life (KOOS-Q). The KOOS-P subscale was used to detect participants’ subjective symptoms and functions.

### Statistical analysis

Demographic characteristics, IFP, and gait parameters were compared between the groups using the non-paired t-test or Mann–Whitney U test. In the OA group, the correlation between the IFP and KOOS scores was examined using either the Pearson or Spearman correlation coefficients, depending on the data distribution. Statistical analyses were performed using SPSS software (v23; IBM, US), with a predetermined significance level of 0.05.

G-power software was used for post-hoc power analysis to detect the correlation between ΔIFP and KOOS-P in the OA group, which indicated a corresponding power of 82.3%.

## Results

### Demographic data and subjective assessment

The demographic data are shown in Table 1. These features were not significantly different between the groups **(**Table 1**).** The knee OA group included several knee OA severities, and almost all the patients had mild to moderate knee OA disease (KLI: 2, II: 10, III: 9, and IV: 5).

### IFP parameters according to MRI evaluation and knee pain in knee OA patients

The area of the IFP was evaluated in patients with knee OA: 634.8 ± 144.9 mm^2^.

The KOOS scores were KOOS-S: 67.5 ± 19.1%, KOOS-P: 58.0 ± 18.9%, KOOS-A: 77.4 ± 17.5%, KOOS-Sp: 37.9 ± 23.9%, and KOOS-Q: 34.1 ± 19.9%.

### Kinetic and kinematic walking parameters

The knee OA group had significantly lower walking speed than the control group (OA: 0.8 ± 0.2 m/s, control: 1.0 ± 0.1 m/s, *p* = 0.002). In the OA group, the range of motion between maximum and minimum knee flexion angle was significantly restricted when compared with that in the control (OA: 8.0 ± 3.3°, control: 10.6 ± 2.5°, *p* = 0.018) **(**Table 2**)**.

**Table 2:** Kinetic and kinematic walking parameters

	Knee OA	Control	*p*-value
Initial knee flexion angle (°)	8.1 ± 5.4	8.7 ± 4.4	0.761
Maximum knee flexion angle (°)	16.1 ± 5.5	19.2 ± 5.3	0.139
Knee flexion range of motion (°)	8.0 ± 3.3	10.6 ± 2.5	0.018
Knee flexion moment (Nm/kg)	0.6 ± 0.2	0.7 ± 0.3	0.178
Gait speed (m/s)	0.8 ± 0.2	1.0 ± 0.1	0.002

OA, osteoarthrosis.

Values are shown as mean ± standard deviation. The significance difference shows *p* < 0.05.

### Thickness of IFP and morphological change

There was no significant difference in IFP thickness in the supine position between the groups (OA: 5.3 ± 1.0 mm, control: 5.1 ± 0.6 mm, *p* = 0.59). During walking, the IFP waveform expanded in the early stance phase and showed different reactions in each knee OA patient **(**Figs. 3 and 4**)**. The minimum and maximum IFP thicknesses were significantly larger in the knee OA group than those in the control group. In contrast, the ΔIFP in the OA group was significantly smaller when compared with that in the control group (OA: 0.9 ± 0.3 mm, control: 1.8 ± 0.2 mm, *p* = 0.001) **(**Table 3**)**.

**Table 3:** Ultrasound parameters of the infrapatellar fat Pat

	Knee OA	Control	*p*-value
Static evaluation			
Thickness of IFP in supine position (mm)	5.3 ± 1.0	5.1 ± 0.6	0.59
Dynamic evaluation			
Minimum thickness of IFP (mm)	4.6 ± 0.7	2.6 ± 0.9	0.001
Maximum thickness of IFP (mm)	5.5 ± 0.7	4.4 ± 0.9	0.001
ΔIFP (mm)	0.9 ± 0.3	1.8 ± 0.2	0.001

OA, osteoarthrosis; IFP, infrapatellar fat pat; ΔIFP, morphological change in IFP showing the difference in IFP value between maximum and minimum.

Values are shown as mean ± standard deviation. The significance difference shows *p* < 0.05.

### Correlation between IFP and biomechanical parameters and subjective outcomes

In patients with knee OA, there was a significant correlation between ΔIFP and KOOS-P scores (*r* = 0.536, *p* = 0.0048). However, other IFP parameters did not significantly correlate with any subjective or biomechanical outcomes **(**Table 4**)**.

**Table 4:** Correlations with IFP parameters

	KOOS-pain	*p*-value
Static evaluation		
Area of IFP (mm^2^)	−0.38	0.053
Thickness of IFP in supine position (mm)	−0.25	0.21
Dynamic evaluation		
Minimum thickness of IFP (mm)	−0.35	0.072
Maximum thickness of IFP (mm)	−0.11	0.585
ΔIFP (mm)	0.54	0.0048

OA, osteoarthrosis; IFP, infrapatellar fat pat; ΔIFP, morphological change in IFP; KOOS, Knee Injury and Osteoarthritis Outcome Score. Values represent the correlation coefficient. Values represent the correlation coefficient and interpret (0.3 ≤ *r* < 0.5) as weak correlation and (0.5 ≤ *r* < 0.7) as some correlation. The significance difference shows *p* < 0.05.

## Discussion

In patients with knee OA, dynamic ultrasonography showed that the ΔIFP during walking was smaller than that in healthy volunteers, and it was correlated with subjective symptom scores, including knee pain, but not with IFP measurements obtained with static evaluation using ultrasound and MRI.

Our data showed that there was a positive correlation between morphological changes and subjective symptom scores. This result indicates that symptomatic patients with knee OA have restricted IFP dynamics. The IFP is known to have a buffer function, owing to flexible morphological changes in the knee joint [8, 9]. Several previous studies reported fibrosis of the IFP in degenerative knee OA [10, 24], which might lead to restricted morphological changes. In this study, patients with knee OA had restricted morphological changes in the IFP, although the knee flexion moment, which implies mechanical stress, did not differ when compared with that of the healthy volunteers. Thus, symptomatic patients with knee OA are prone to declining knee buffer function under mechanical stress, which could cause substantial stress within the knee joint. The results of these previous studies and our data might explain the restricted dynamics of the IFP and reflect an abnormal mechanopathology in patients with symptomatic OA.

The data on IFP parameters included the area and thickness at static evaluation in the supine position, and there was no significant difference between the groups or correlation with subjective knee outcomes. Thus, the morphological features of the IFP did not reflect the patient’s symptoms. This is in line with previous studies that investigated the effects of IFP edema or fibrogenesis on symptomatic knee OA [13, 25]. The IFP has unique features and changes its structure in response to the situation, such as motion-induced muscle contraction and weight-bearing [21, 26]. In this study, the participants demonstrated symptoms of knee pain during daily activities that persisted for months, implying repeated exposure of their knees to mechanical stress. Morphological changes in the IFP have recently been reported to correlate with knee pain during squatting [19]. Therefore, the morphological change in the IFP under dynamic conditions could be sensitively detected in symptomatic patients when compared with static conditions.

Some patients report knee pain during activities of daily living under mechanical stress in clinical settings. However, the mechanical stress during walking reflecting the knee flexion moment was not different between the groups and was not correlated with knee pain. This points to an inconsistent background in mechanopathological conditions, such as OA. One explanation could be different adjustment functions of mechanical stress within the knee joint. Dynamic ultrasound evaluation elucidates the reaction of the soft tissue to mechanical stress and its association with knee pain [27–29], and the present study demonstrated a restricted morphological IFP during walking. Therefore, elucidation of the mechanopathology in symptomatic knee OA is emphasized to consider not only mechanical stress during motion but also dynamic morphological changes within the joint using dynamic evaluation.

This study had several limitations. First, this study had a small sample size, which raised concerns that analyses such as knee OA severity and knee alignments could not be performed. Second, the evaluation of knee pain did not include knee pain in the knee compartment, and it remains unknown whether anterior knee pain occurs. Third, this study found that a restricted IFP was a feature of symptomatic knee OA. However, the design of this study did not allow us to determine the consequences of the IFP causing knee pain during walking. Fourth, knee OA patients had differential gait speeds when compared with healthy volunteers. Although there was no significant correlation between gait speed and the IFP, our data and design could not directly rule out the effect of gait speed on the IFP. Future studies need to add a detailed clinical evaluation and include a sufficient sample size in the cohort design.

## Conclusion

Restricted morphological changes in the IFP during walking could be more sensitively detected with dynamic evaluation in patients with symptomatic knee OA as opposed to static evaluation.

## Data Availability

The data that support the findings of this study are available from the corresponding author upon reasonable request.

## References

[1] Steenkamp W, Rachuene PA, Dey R, et al. The correlation between clinical and radiological severity of osteoarthritis of the knee. SICOT-J. 2022;8:14. 35389338 10.1051/sicotj/2022014 PMC8988866

[2] Felson DT. Osteoarthritis as a disease of mechanics. Osteoarthritis Cartilage. 2013;21:10–5. 23041436 10.1016/j.joca.2012.09.012 PMC3538894

[3] Dye SF, Vaupel GL, Dye CC. Conscious neurosensory mapping of the internal structures of the human knee without intraarticular anesthesia. Am J Sports Med. 1998;26:773–7. 9850777 10.1177/03635465980260060601

[4] Scapinelli R. Vascular anatomy of the human cruciate ligaments and surrounding structures. Clin Anat. 1997;10:151–62. 9135883 10.1002/(SICI)1098-2353(1997)10:3<151::AID-CA1>3.0.CO;2-X

[5] Bohnsack M, Meier F, Walter GF, et al. Distribution of substance-P nerves inside the infrapatellar fat pad and the adjacent synovial tissue: a neurohistological approach to anterior knee pain syndrome. Arch Orthop Trauma Surg. 2005;125:592–7. 15891922 10.1007/s00402-005-0796-4

[6] Dragoo JL, Phillips C, Schmidt JD, et al. Mechanics of the anterior interval of the knee using open dynamic MRI. Clin Biomech Bristol Avon. 2010;25:433–7. 10.1016/j.clinbiomech.2010.01.011 20189271

[7] Okita Y, Oba H, Miura R, et al. Movement and volume of infrapatellar fat pad and knee kinematics during quasi-static knee extension at 30 and 0° flexion in young healthy individuals. Knee. 2020;27:71–80. 31918962 10.1016/j.knee.2019.10.019

[8] Bohnsack M, Wilharm A, Hurschler C, et al. Biomechanical and kinematic influences of a total infrapatellar fat pad resection on the knee. Am J Sports Med. 2004;32:1873–80. 15572315 10.1177/0363546504263946

[9] Stephen JM, Sopher R, Tullie S, et al. The infrapatellar fat pad is a dynamic and mobile structure, which deforms during knee motion, and has proximal extensions which wrap around the patella. Knee Surg Sports Traumatol Arthrosc. 2018;26:3515–24. 29679117 10.1007/s00167-018-4943-1

[10] Fontanella CG, Belluzzi E, Rossato M, et al. Quantitative MRI analysis of infrapatellar and Suprapatellar fat pads in normal controls, moderate and end-stage osteoarthritis. Ann Anat. 2019;221:108–14. 30292837 10.1016/j.aanat.2018.09.007

[11] Cowan SM, Hart HF, Warden SJ, et al. Infrapatellar fat pad volume is greater in individuals with patellofemoral joint osteoarthritis and associated with pain. Rheumatol Int. 2015;35:1439–42. 25782586 10.1007/s00296-015-3250-0

[12] Han W, Cai S, Liu Z, et al. Infrapatellar fat pad in the knee: is local fat good or bad for knee osteoarthritis? Arthritis Res Ther. 2014;16:R145. 25008048 10.1186/ar4607 PMC4227074

[13] Steidle-Kloc E, Culvenor AG, Dörrenberg J, et al. Relationship between knee pain and infrapatellar fat pad morphology: A Within- and between-Person analysis from the osteoarthritis initiative. Arthritis Care Res. 2018;70:550–7. 10.1002/acr.23326 28704603

[14] Hart HF, Culvenor AG, Patterson BE, et al. Infrapatellar fat pad volume and Hoffa-synovitis after ACL reconstruction: association with early osteoarthritis features and pain over 5 years. J Orthop Res. 2022;40:260–7. 33458849 10.1002/jor.24987

[15] Ishii Y, Nakashima Y, Ishikawa M, et al. Dynamic ultrasonography of the medial meniscus during walking in knee osteoarthritis. Knee. 2020;27:1256–62. 32711889 10.1016/j.knee.2020.05.017

[16] Ishii Y, Ishikawa M, Nakashima Y, et al. Knee adduction moment is correlated with the increase in medial meniscus extrusion by dynamic ultrasound in knee osteoarthritis. Knee. 2022;38:82–90. 35930897 10.1016/j.knee.2022.07.011

[17] Favero M, El-Hadi H, Belluzzi E, et al. Infrapatellar fat pad features in osteoarthritis: a histopathological and molecular study. Rheumatol Oxf Engl. 2017;56:1784–93. 10.1093/rheumatology/kex287 28957567

[18] Fontanella CG, Belluzzi E, Pozzuoli A, et al. Mechanical behavior of infrapatellar fat pad of patients affected by osteoarthritis. J Biomech. 2022;131:110931. 34972018 10.1016/j.jbiomech.2021.110931

[19] Shiraishi R, Ueda S. Relationship between the change in infrapatellar fat pad thickness assessed using ultrasonography and anterior knee pain on squatting after anterior cruciate ligament reconstruction. J Med Ultrason. 2001. 2023;50:237–43. 10.1007/s10396-023-01300-3 PMC11018648 36961646

[20] Steadman JR, Dragoo JL, Hines SL, et al. Arthroscopic release for symptomatic scarring of the anterior interval of the knee. Am J Sports Med. 2008;36:1763–9. 18753680 10.1177/0363546508320480

[21] Naredo E, Canoso JJ, Yinh J, et al. Dynamic changes in the infrapatellar knee structures with quadriceps muscle contraction. An in vivo study. Ann Anat. 2021;235:151663. 33387611 10.1016/j.aanat.2020.151663

[22] Okita Y, Miura R, Morimoto M, et al. Three-dimensional volume and shape of the infrapatellar fat pad during quasi-static knee extension from 30° to 0°: comparisons of patients with Osteoarthritic knees and young, healthy individuals. J Phys Ther Sci. 2023;35:507–14. 37405182 10.1589/jpts.35.507 PMC10315202

[23] Okinaka R, Ishii Y, Nakashima Y, et al. Morphological changes in the infrapatellar fat pad during walking detected by dynamic ultrasound in healthy volunteers. Cureus. 2024;16:e66738. 39268287 10.7759/cureus.66738 PMC11392513

[24] Chang J, Liao Z, Lu M, et al. Systemic and local adipose tissue in knee osteoarthritis. Osteoarthritis Cartilage. 2018;26:864–71. 29578044 10.1016/j.joca.2018.03.004

[25] Bohnsack M, Klages P, Hurschler C, et al. Influence of an infrapatellar fat pad edema on patellofemoral biomechanics and knee kinematics: a possible relation to the anterior knee pain syndrome. Arch Orthop Trauma Surg. 2009;129:1025–30. 17053945 10.1007/s00402-006-0237-z

[26] Katayama N, Noda I, Fukumoto Y, et al. Effects of isometric contraction of the quadriceps on the hardness and blood flow in the infrapatellar fat pad. J Phys Ther Sci. 2021;33:722–7. 34658513 10.1589/jpts.33.722 PMC8516604

[27] Ishii Y, Ishikawa M, Nakashima Y, et al. Dynamic ultrasound reveals the specific behavior of the medial meniscus extrusion in patients with knee osteoarthritis. BMC Musculoskelet Disord. 2023;24:272. 37038148 10.1186/s12891-023-06361-6 PMC10084641

[28] Ishii Y, Ishikawa M, Nakashima Y, et al. Unique patterns of medial meniscus extrusion during walking and its association with limb kinematics in patients with knee osteoarthritis. Sci Rep. 2023;13:12513. 37532866 10.1038/s41598-023-39715-0 PMC10397274

[29] Ishii Y, Ishikawa M, Nakashima Y et al. Dynamic response of medial meniscus extrusion to the lateral wedge insole is correlated with immediate pain reduction in knee osteoarthritis patients: real-time ultrasonographic study. J Med Ultrason. 2001. 2022; 49:731–8. 10.1007/s10396-022-01234-2 35790646

